# Supplementary material for: A prospective model of the potential clinical and economic impact of cervical cancer screening supported by a mobile phone app
Source: PLoS One. 2025 Jan 31;20(1):e0316001. doi: 10.1371/journal.pone.0316001 (PMC11785268; doi:10.1371/journal.pone.0316001)
Supplement: S1 Appendix — (DOCX) [file pone.0316001.s001.docx]

**Supporting Information**

**Appendix: Screening visit and follow-up visit structure for prospective clinico-economic model**

***Clinical pathway and outcomes***

As reflected in the clinical workflow diagram in Figure 1, women who were eligible for cervical cancer screening during visit 1, were required to agree to participate in the study, sign study consent forms to undergo a pelvic examination, and undergo collection of cervical cells for primary screening with cobas®4800 HPV Test and VIA/VILI. At the same visit, women who had an Android smartphone available on the day of screening were invited to participate in the evaluation of mobile app and be randomly assigned to the intervention or control group. The patients downloaded the app and registered with their phone number and year of birth. All samples and results were scanned into the mobile application using a barcode to link the specimen and the app. VIA results were available immediately whereas HPV results were available after 14 days. Those who are VIA-positive with visual suspicion of cancer are immediately biopsied whereas those who are VIA-negative with no suspicion of cancer get their samples taken for HPV screening.

During visit 2, all women who were HPV-positive by cobas® 4800 HPV Test or VIA/VILI positive were referred for a standardized colposcopy examination for diagnosis. During colposcopy, biopsies were obtained from any abnormally appearing areas to ascertain neoplastic outcomes and to direct treatment as required. The intervention group was assisted to scan the barcode associated with their biopsy sample and can view their result with the app when available. The control group was provided with an appointment in two weeks to receive their biopsy test results. The intervention group received all test results through the app and received digital reminders to adhere to follow-up visits. All women who had cancer or high-grade cervical lesions (CIN2+) were encouraged to receive treatment.

The goal of appointment reminders is to ensure engagement with the screening cycle and avoid loss to follow-up. The clinic schedules appointment reminders every other day during the first two weeks after receiving the visit and every day after two weeks. The reminders are stopped when either: the test/ treatment was scanned or four weeks after receiving the result without further scan (loss to follow-up). For regular reminders, the goal is to ensure client engagement in the screening program and routine visits. The schedule for regular reminders is once after receiving a visit, six months before the next routine visit, one month before the next routine visit, and one week before the next routine visit.

We used expert elicitation techniques with SMEs involved in screening and treatment for cervical cancer to obtain information about clinical and behavioral parameters based on their clinical experience and professional judgment. Additionally, a simplified time-and-motion study was undertaken in two oncology clinics participating in the randomized study by observing study-related visits for 15 patients throughout clinical workflow processes for visits 1 and 2. Finally, key informant interviews were conducted with administrative and clinical staff in four study sites to understand patient volume and patient treatment pathway issues for cervical cancer screening and follow-up care. The focus was on standard patient encounters in clinics to generate resource use and cost estimates for cervical cancer-related visits for testing (screening) and visits for pre-cancer and cancer-related procedures. Additional details on methods used for the time-and-motion and expert elicitation studies are available from the authors. All prospective data-collection activities received approval by the clinical study’s original IRB in Kenya.

***Clinical outcomes and treatments***

Key outcomes from visit 1 included same-day VIA results (VIA-positive with visual suspicion of cancer and VIA-negative with no suspicion of cancer). Patients would have a VIA-/HPV- with the following options (rescreen in five years if HIV-; rescreen within two years if HIV+ and return for annual routine testing). Patients were also given reminders for appointments as well as educational messages. Those who were VIA+/HPV- follow up with clinic; those who were HPV+ non 16/18 genotypes were to follow up with clinic and continue to get education/explanation of next steps. For those with VIA+ or VIA- results with positive HPV 16/18 or HPV non-16/18 results, there was a follow-up immediately with the clinic to schedule a colposcopy/biopsy visit. These patients received reminders to return for colposcopy until the nurse scanned the colposcopy visit outcome. Our model allowed for a proportion of patients to receive colposcopy in “lower-risk test result groups,” adjusting from the base-case value of 100%.

At visit 2, all patients were scheduled to receive a colposcopy and if they needed a biopsy, and/or pre-cancer treatment. For those who underwent colposcopy after visit 1 results, there were three scenarios for the results from the colposcopy. Where the colposcopy was normal (they would be required to rescreen at three years if HIV- or rescreen every one year if HIV+), they were also to get reminders to return for annual routine tests and educational messages through the app. Those patients with an abnormal colposcopy would either have a biopsy collected or receive pre-cancer treatment.

The biopsy results were classified according to the CIN status. For CIN1 or less, the patients were advised to be rescreened in three years if HIV negative or get rescreened within one year if HIV positive. These patients were also given regular reminders for an annual routine visit and educational messages. Those classified as CIN2+ followed -up immediately with the clinic for appropriate treatment (cryotherapy, thermocoagulation, or LEEP), and are sent reminders until treatment is provided. Those with confirmed cancer follow up immediately with the clinic until treatment (chemotherapy/radiotherapy) is started.

Visit 3 treatment outcomes were diagnosis and/or pre-cancer/cancer treatment. Following the biopsy results the goal was to initiate treatment immediately for pre-cancer (CIN2+) and cancer patients and ensure patients followed up with subsequent visits and appointments until treatment was completed.
